# *Bacillus cereus* Biofilms—Same, Only Different

**DOI:** 10.3389/fmicb.2016.01054

**Published:** 2016-07-07

**Authors:** Racha Majed, Christine Faille, Mireille Kallassy, Michel Gohar

**Affiliations:** ^1^Micalis Institute, INRA, AgroParisTech, CNRS, Université Paris-SaclayJouy-en-Josas, France; ^2^Unité de Recherche Technologies et Valorisation Alimentaire, Laboratoire de Biotechnologie, Université Saint-JosephBeirut, Lebanon; ^3^UMR UMET: Unité Matériaux et Transformations, Centre National de la Recherche Scientifique, Institut National de la Recherche Agronomique, Université de LilleVilleneuve d'Ascq, France

**Keywords:** *Bacillus*, *cereus*, *thuringiensis*, *anthracis*, biofilm, ecology, regulation, food

## Abstract

*Bacillus cereus* displays a high diversity of lifestyles and ecological niches and include beneficial as well as pathogenic strains. These strains are widespread in the environment, are found on inert as well as on living surfaces and contaminate persistently the production lines of the food industry. Biofilms are suspected to play a key role in this ubiquitous distribution and in this persistency. Indeed, *B. cereus* produces a variety of biofilms which differ in their architecture and mechanism of formation, possibly reflecting an adaptation to various environments. Depending on the strain, *B. cereus* has the ability to grow as immersed or floating biofilms, and to secrete within the biofilm a vast array of metabolites, surfactants, bacteriocins, enzymes, and toxins, all compounds susceptible to act on the biofilm itself and/or on its environment. Within the biofilm, *B. cereus* exists in different physiological states and is able to generate highly resistant and adhesive spores, which themselves will increase the resistance of the bacterium to antimicrobials or to cleaning procedures. Current researches show that, despite similarities with the regulation processes and effector molecules involved in the initiation and maturation of the extensively studied *Bacillus subtilis* biofilm, important differences exists between the two species. The present review summarizes the up to date knowledge on biofilms produced by *B. cereus* and by two closely related pathogens, *Bacillus thuringiensis* and *Bacillus anthracis*. Economic issues caused by *B. cereus* biofilms and management strategies implemented to control these biofilms are included in this review, which also discuss the ecological and functional roles of biofilms in the lifecycle of these bacterial species and explore future developments in this important research area.

## Introduction

*Bacillus cereus* is a large, Gram-positive bacterium which produces spores and displays a peritrichous flagellation. Soil has long been considered to be the natural habitat of this species, although its spores can be isolated from various materials, such as invertebrates, plants, or food (Sneath, 1986). Recently, the ecological niches of *B. cereus* were suggested to include insects and nematodes guts (Jensen et al., 2003; Ruan et al., 2015), or plant roots (Ehling-Schulz et al., 2015). The high diversity of *B. cereus* habitats is reflected by the genetic polymorphism of this species (Helgason et al., 2004), and is illustrated by the existence of probiotic (Cutting, 2011) as well as pathogenic strains. *B. cereus* is indeed one of the most frequent agent of food poisoning outbreaks, which symptoms can be either emetic or diarrheal. Emetic strains of *B. cereus* can secrete in the food a highly toxic and heat-stable Non-ribosomal cyclic peptide which can withstand cooking temperatures and induce, when ingested, vomitic symptoms (Ehling-Schulz et al., 2015). For diarrheal strains, according to the current model of *B. cereus*-induced diarrheal gastroenteritis, spores contained in the food are ingested by the host and germinate within the intestine, where vegetative cells can grow and produce enterotoxins. Three enterotoxins (Hbl, Nhe, and CytK) can be secreted by *B. cereus* (Stenfors Arnesen et al., 2008). In addition to enterotoxins, *B. cereus* can produce several other toxins (hemolysins HlyI and HlyII) and degradative enzymes (phospholipases and proteases), which are either secreted or directed to the cell-surface, and which are controlled, for most of them, by the PlcR transcriptional activator (Gohar et al., 2008). PlcR is one of the numerous *B. cereus* quorum-sensing systems, which, together with a great number of chromosomally-encoded sensors and regulators (De Been et al., 2006), make the bacterium highly responsive to environmental changes and give it the ability to adapt to diverse conditions. The adaptative properties of *B. cereus* is also a consequence of the presence, within the bacterium, of a number of plasmids, which size is in the 2–500 kb range. *Bacillus thuringiensis* and *Bacillus anthracis*, for instance, are two species of the *B. cereus* group *sensu lato* which differ from *B. cereus sensu stricto* mainly by the presence of megaplasmids carrying genes encoding toxins specifically active against, respectively, invertebrates or mammals.

**Figure 1:**
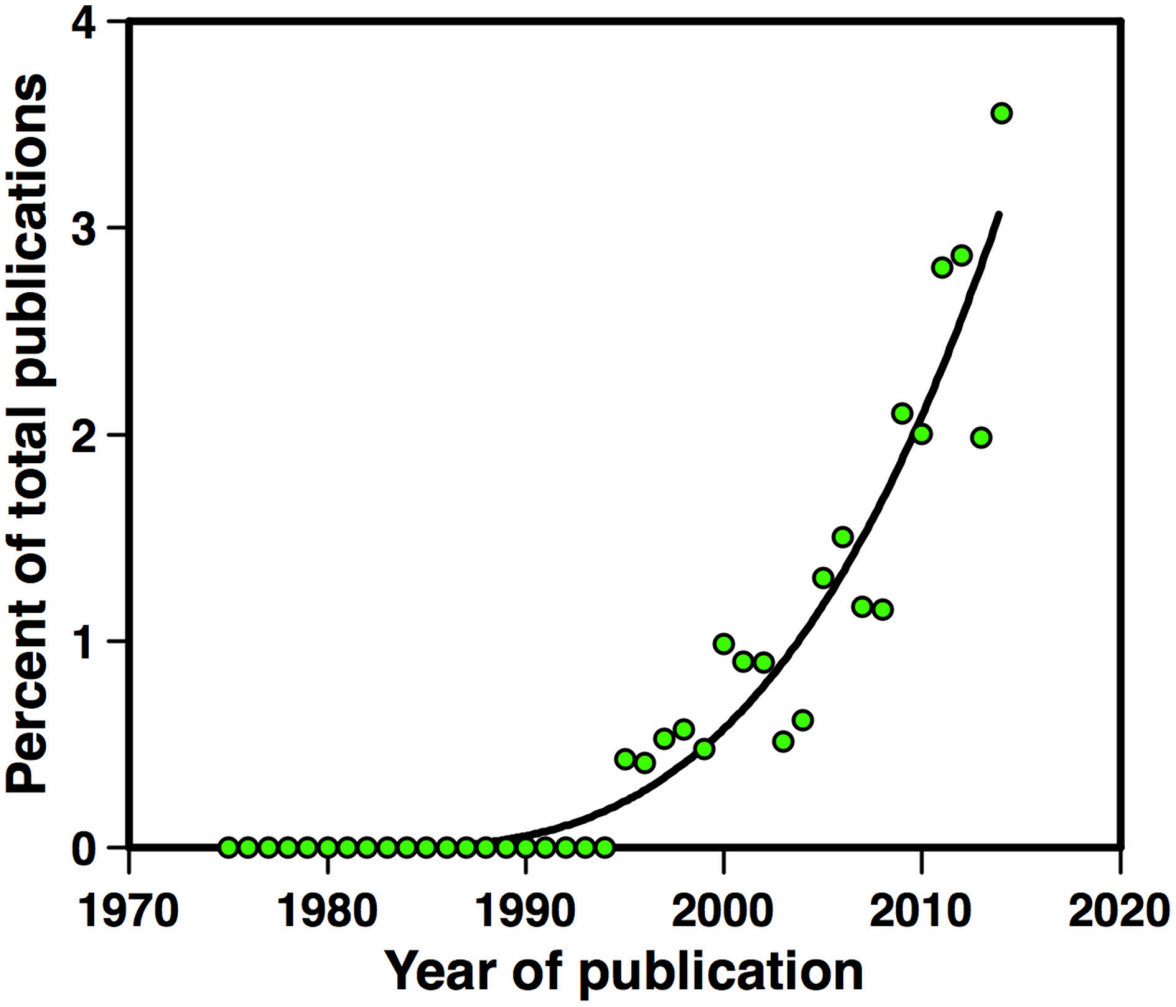
**Number of articles published between 1975 and 2015 on *B. cereus* biofilms**. Articles published on *B. cereus, B. thuringiensis*, or *B. anthracis* biofilms, in percent of the total number of articles published on the same species.

*B. cereus, B. thuringiensis*, and *B. anthracis* (called hereafter *B. cereus sensu lato*) are all able to produce biofilms. In most isolates of these species, biofilms are found as floating pellicles, but can also stick on immerged abiotic surfaces or even be present on living tissues. These complex communities are likely to be a key element in the ability of *B. cereus* to colonize different environments. Together with spores, they confer to the bacterium a high resistance to various stresses and a high adhesive capacity on various substrates, including stainless steel, a material widely used in the food processing lines. In these facilities, *B. cereus* can persist for long durations and can even withstand sanitization procedures. The exponential increase in the number of articles published on *B. cereus* biofilms (Figure 1) illustrates the rising interest of the scientific community for this subject. Indeed not only are biofilms a key issue in *B. cereus* life, they also display interesting specificities. Although some of the molecular mechanisms involved in biofilm formation and in its regulation are shared with *Bacillus subtilis*—a saprophytic bacterium extensively studied for biofilm formation—striking differences exists between the two species regarding the biofilm structure, the effectors of matrix formation and the regulation pathways controlling them.

In the last decade, a considerable knowledge has been accumulated in a wide area of research regarding biofilm formation in *B. cereus sensu lato*. The aim of this review is to stress a panoramic view of the current knowledge, from the molecular mechanisms involved in biofilms formation in the three species to the functions and roles of these multicellular structures in the bacterium life, including pathogenesis and food industry contamination. From this panoramic view, we expect to draw the most promising incoming research developments and to address some intriguing questions, such as why has *B. anthracis*, a lethal and capsulated pathogen, kept the ability to produce biofilms. This review will also highlight the variety and prevalence of biofilm formation in the three species and will point, when necessary, to similarities and differences with *B. subtilis*.

## Molecular and physiological aspect

The molecular and physiological aspects of biofilm formation discussed here include the various extracellular macromolecules produced by the bacterium and specifically required for the biofilm matrix, cellular elements involved in biofilm formation such as flagella or cell-surface proteins, and the complex regulation network controlling biofilm formation and connecting it to other cellular functions. Also included in this part of the review is phenotypic heterogeneity within the biofilm, a field of growing interest since it is strongly involved in the bacterial survival in changing environments, and the role of mobile genetic elements in biofilm formation.

### The biofilm matrix

Biofilms are usually embedded in a self-produced matrix whose structural elements are exopolysaccharides, proteins and DNA (Flemming and Wingender, 2010). *B. cereus* is no exception to this rule and its matrix contains the three components. In *B. subtilis*, most of the structural exopolysaccharides required for biofilm formation are synthetized by the products of the *epsA-O* operon (Branda et al., 2001; Kearns et al., 2005). Deletion of *epsA-O* leads to a Non-structured and fragile biofilm pellicle (Lemon et al., 2008). An *eps* locus similar to *epsA-O* is found in bacteria of the *cereus* group (Ivanova et al., 2003; Gao et al., 2015). This similarity is supported by the presence, within the locus, of an anti-termination RNA element named EAR, found only in *epsA-O* and in the *eps* locus of the *cereus* group (Irnov and Winkler, 2010). However, deletion of the *B. cereus eps* locus does not affect biofilm formation (Gao et al., 2015), despite the presence of polysaccharides in the *B. cereus* biofilm matrix (Houry et al., 2012), whose origin therefore remains unknown.

The *B. subtilis* biofilm matrix also contains the three structural proteins TasA, TapA, and BslA (Vlamakis et al., 2013). BslA (Biofilm surface layer) forms a hydrophobic envelope surrounding the biofilm (Hobley et al., 2013) while TasA assembles into amyloid-like fibers attached to the cell wall by TapA, resulting in a fiber network strengthening the biofilm (Romero et al., 2011). In *B. subtilis, tapA*, and *tasA* are included in the *tapA-sipW-tasA* operon, where *sipW* codes for a signal peptidase, which releases the two proteins TapA and TasA into the extracellular milieu. There is no paralog of *bslA* or *tapA* in the *B. cereus* genome, but *tasA* have two paralogs. One is *tasA*, included in the *sipW-tasA* operon, and the other is *calY*, which is located next to *sipW-tasA* (Caro-Astorga et al., 2015). TasA and CalY are both involved in the production of fibers which can be observed by electron microscopy, and the deletion of their genes or of *sipW* leads to biofilm defects similar to the ones reported in *B. subtilis* (Caro-Astorga et al., 2015).

The extracellular DNA (eDNA) contained in the *B. cereus* biofilm matrix was shown to be produced specifically in biofilms and was reported to be required for adhesion on polystyrene or glass surfaces (Vilain et al., 2009). Its origin remains unknown but might be related to programmed cell death (Abee et al., 2011). However, in planktonic cultures of *B. subtilis*, the production of eDNA is not a consequence of cell-lysis but requires both competence genes and the Opp oligopeptide permease, and is involved in horizontal gene transfer (Zafra et al., 2012). Other bacterial species, including the Gram-positive bacteria *Staphylococcus aureus* and *Streptococcus pneumonia*, also require eDNA for biofilm formation (Whitchurch et al., 2002; Moscoso et al., 2006; Izano et al., 2008). Possible interactions between the eDNA and other consituents of the biofilm matrix have not yet been investigated, neither has the mechanism or the regulation of eDNA production in biofilms.

### Role of flagella

Flagella are cell-surface structures extending far away the bacterial cell. In *B. cereus*, they are not required for adhesion to glass (Houry et al., 2010), but flagellar motility is involved in biofilm formation through 4 mechanisms. First, motility is a key element of biofilm formation when the bacterium must reach by its own (in static conditions) suitable places for biofilm formation (Houry et al., 2010), at the air-liquid interface. The suppression of motility in a strain which forms biofilms at the air-liquid interface resulted in the formation of submerged biofilms (Hayrapetyan et al., 2015b). Secondly, motile bacteria within the biofilm create channels in the matrix, leading to an increase in nutrients exchange and, conversely, favoring the penetration of toxic substances (Houry et al., 2012). Thirdly, motile planktonic bacteria can enter the biofilm and increase its biomass (Houry et al., 2010, 2012). Fourthly, motile bacteria located at the edge of the growing biofilm extend the surface covered by this structure, resulting in colony spreading (Houry et al., 2010). Although flagellin transcription decreases continuously with biofilm age (Houry et al., 2010), the biofilm bacterial population is heterogeneous and includes a fraction of motile bacteria (Houry et al., 2012) which, in *B. subtilis*, is located at the edge of the colony (Vlamakis et al., 2008).

### Cell-surface properties

*B. cereus* cells in biofilm differ from their planktonic counterparts regarding their cell-surface properties. For example, the structure of the secondary cell wall polymer (SCWP), a polysaccharide linked to the peptidoglycan by phospho-diester linkages, was shown to vary during biofilm aging in *B. cereus* (Candela et al., 2011). Since SLH (S-layer homology) domain-containing proteins bind to the SCWP, changes in the SCWP structure might result in changes in the proteins displayed on the cell-surface, and possibly involved in the adaptation of the bacterium to its environment. Within these SLH-proteins are autolysins, whose variation during biofilm growth might lead to changes in the bacterial chain length. Similarly, a cell-surface peptidase (CwpFM) involved in autolysis was shown to play a role in biofilm formation, possibly because this autolysin can modulate the length of bacterial chains and consequently act on the motility of the bacterium (Tran et al., 2010).

### Regulation networks

**Figure 2:**
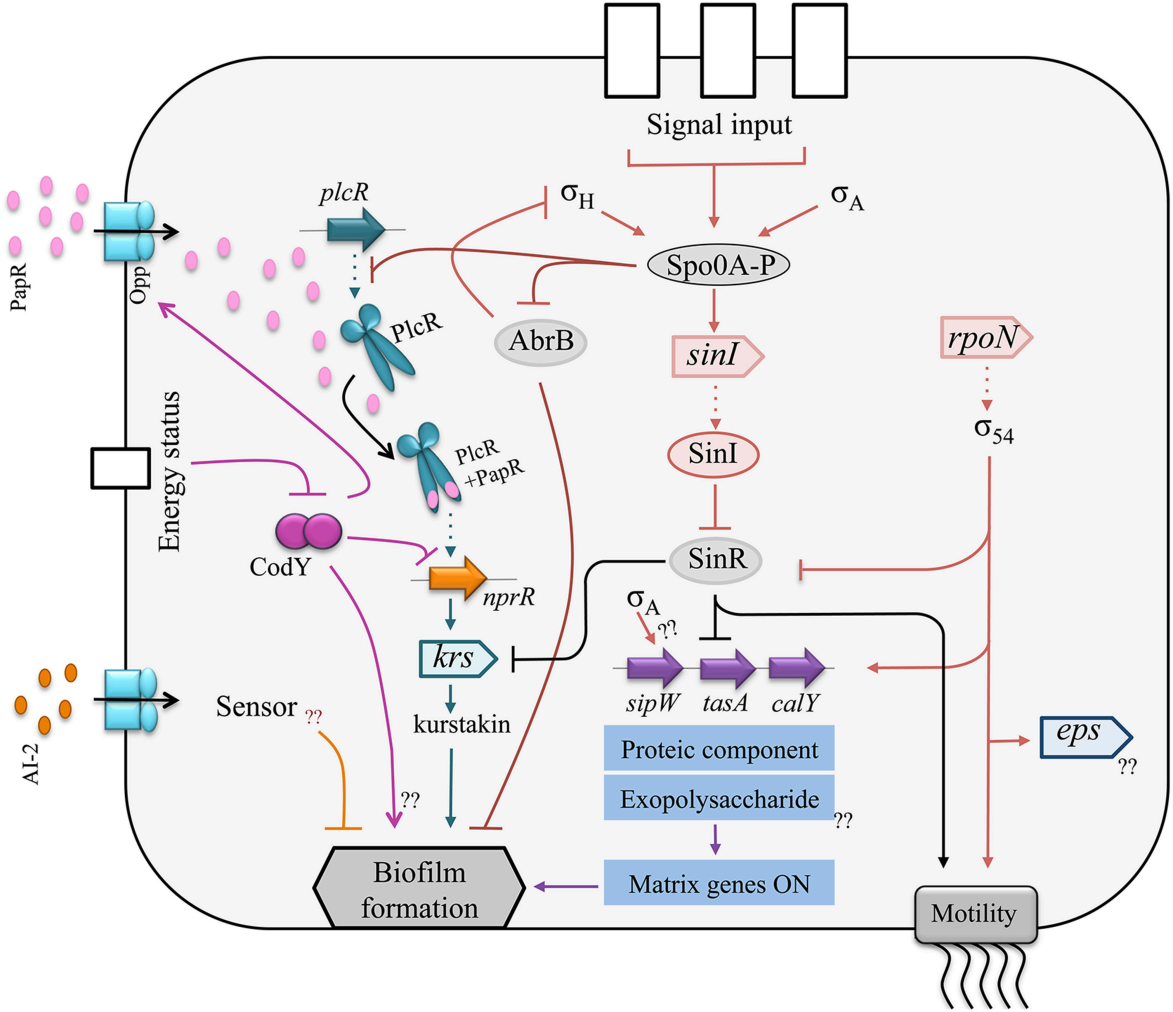
**Schematic representation of the regulatory network controlling biofilm formation in *B. cereus.*** *Circles symbolize proteins, triangles symbolize open reading frames (ORFs)*. Arrows indicate activation and blunt lines indicate repression. Dotted arrows represent transcription. The protein component of the matrix is encoded by the *sipW*-*tasA* operon and by *calY* which promoters are activated by σ54 and repressed by SinR. SinR is antagonized by SinI. The transcription of *sinI* is activated by the master regulator of sporulation Spo0A. Furthermore, Spo0A downregulates the regulator AbrB, resulting in biofilm formation. Several quorum sensing systems are involved in biofilm formation. The regulator PlcR activates the transcription of *nprR*. NprR promotes kurstakin production, which itself promotes biofilm formation. The autoinducer AI-2 plays an inhibitory effect on biofilm formation.

The regulation network controlling *B. cereus* biofilm formation shows a combination of similarities and differences with *B. subtilis*. In *B. cereus sensu lato, sipW, tasA*, and *calY* transcriptions are repressed by the SinR regulator (Pflughoeft et al., 2011), which controls biofilm formation (Fagerlund et al., 2014) as for *B. subtilis*. SinR is antagonized by SinI and, in both species, deletion of SinI leads to the absence of biofilm and to hypermotility while the reverse phenotype (biofilm overproduction, no motility) is obtained upon deletion of SinR (Kearns et al., 2005; Fagerlund et al., 2014; Figure 2). Consequently, the SinI/SinR anti-repressor/repressor pair is likely to act as a switch between biofilm formation and swimming motility in *B. cereus* or *B. thuringiensis* as it does in *B. subtilis*. In addition, Spo0A is required for biofilm formation in *B. thuringiensis* and in *B. subtilis*, and AbrB represses biofilm formation in both species (Hamon and Lazazzera, 2001; Fagerlund et al., 2014).

However, the SinR regulon also displays important differences in the two species: the *B. subtilis epsA-O*, but not the *B. thuringiensis eps*, is included in this regulon. Conversely, the production of kurstakin, a lipopeptide biosurfactant, is controlled by SinR in *B. thuringiensis* while surfactin, a *B. subtilis* lipopeptide, is not in the SinR regulon. Kurstakin is also included in the NprR necrotrophic regulon required for survival in the insect cadaver (Dubois et al., 2012), and the hemolysin Hbl, controlled by SinR in *B. thuringiensis* (Fagerlund et al., 2014), is included in the PlcR virulence regulon of this species (Gohar et al., 2008). Other differences, in addition to the SinR regulon, exist between *B. subtilis* and *B. cereus sensu lato* for the regulation of biofilm formation. The AI2 autoinducer represses biofilm formation in *B. cereus* (Auger et al., 2006), but induces biofilm formation in *B. subtilis* (Duanis-Assaf et al., 2015), and the DegU regulator, which controls biofilm formation in *B. subtilis* (Kobayashi, 2007b; Cairns et al., 2014), has no homolog in *B. cereus*.

**Figure 3:**
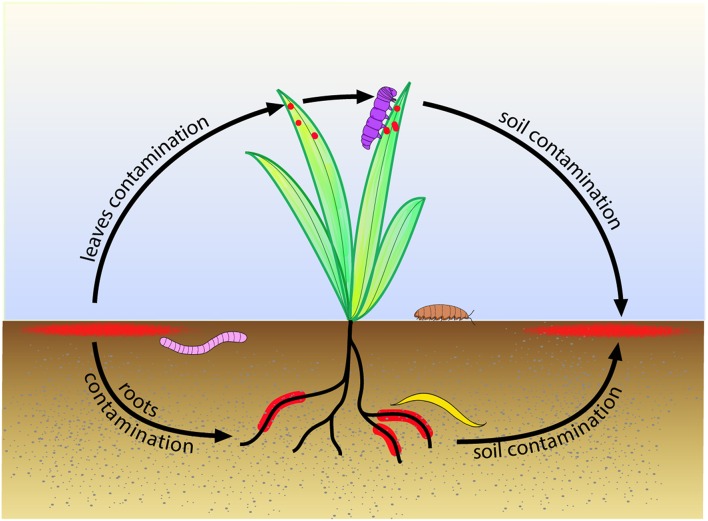
**Suggested model for biofilm role in the life cycle of *B. cereus* and *B. thuringiensis* in the environment**. Biofilms (in red) growing in the topsoil contaminate the roots and leaves of plants. Earthworm (in pink) feeding on soil organic matter, nematodes (in yellow) feeding on plant roots, caterpillar (in purple) feeding on plant leaves, or isopodes (in brown) feeding on plant debris, ingest bacteria, which can then grow as biofilms in their gut. The invertebrates move further in the environment and, upon death, contaminate back the topsoil, giving birth to a new cycle.

In *B. thuringiensis*, there is an interaction between biofilm formation, virulence and necrotrophism in insects (Figure 3), since PlcR promotes NprR transcription (Dubois et al., 2013), which positively controls kurstakin transcription (Dubois et al., 2012), which, in turn, promotes biofilm formation (Gélis-Jeanvoine et al., 2016). In *B. cereus* strain ATCC14579, PlcR was reported to repress biofilm formation (Hsueh et al., 2006), which is in disagreement with these observations. The disruption of *nprR* by a transposon in strain ATCC14579, and therefore the shutdown of the necrotrophic regulon, can explain this discrepancy. For the same reason, the regulator CodY was reported, either to repress biofilm formation in the *B. cereus* ATCC14579 strain (Lindbäck et al., 2012), or to promote biofilm formation in the *B. cereus* UW101C strain (Hsueh et al., 2008). CodY is a regulator sensing the energy and the nutrient state of the bacterial cell (Sonenshein, 2005). It promotes PlcR transcription in stationary phase (Frenzel et al., 2012; Lindbäck et al., 2012) by inducing the production of a transporter required for the import of the PlcR-activating peptide PapR (Slamti et al., 2015), and represses NprR transcription in exponential phase (Dubois et al., 2013). Therefore, the expected effect of CodY on biofilm formation, if this phenotype is induced in early stationary phase, should rather be positive. The connection between biofilm formation and virulence is mediated by another regulator in *B. cereus*. In this species, Sigma 54 (RpoN) promotes the transcription of virulence factors, *eps* genes and flagellins (Hayrapetyan et al., 2015b). These interconnections are an indication that biofilms could be involved in the pathogenic, commensal or necrotrophic lifestyles of *B. cereus sensu lato*.

### Heterogeneity in the biofilm

The limited diffusion of nutrients and signal molecules within the biofilm matrix creates micro-environments and local quorum-sensing states, resulting in a heterogeneous spatial distribution of bacteria in different physiological states. This heterogeneity has been described in several species, including *B. subtilis*, where vegetative cells, sporulating cells, and matrix-producing cells co-exist with different spatial localizations (Vlamakis et al., 2008). In *B. thuringiensis*, motile vegetative cells make from 0.1 to 1% of the total biofilm population and could be beneficial to the whole community by creating channels within the biofilm matrix (Houry et al., 2012). In the same species, in a 48 h-aged biofilm, about 15% of the cells express the enterotoxin Hbl (Fagerlund et al., 2014) which, if it accumulates within the matrix, could make the biofilm a toxic patch-like structure when formed on host tissues. Actually, the biofilm matrix of strains ATCC14579 and ATCC10987 contains the enterotoxins Hbl and Nhe, a collagenase, the phospholipases PI-PLC and sphingomyelinase, and the immune inhibitor protease InhA1, all being virulence factors (Karunakaran and Biggs, 2011). Genes expression heterogeneity within the *B. thuringiensis* biofilm evolves with time, from 24 to 72 h, and shows a decrease in the proportion of bacteria expressing virulence genes, an increase in the proportion of bacteria expressing necrotrophic genes, and a constant proportion of sporulating cells (about 15%; Verplaetse et al., 2015). Interestingly, necrotrophic bacteria arouse mainly from cells which have previously expressed virulence genes. In a sporulating medium, only necrotrophic and sporulating bacteria were observed in the biofilm (Verplaetse et al., 2016).

### Mobile genetic elements

Plasmids were shown to be involved in biofilm formation in a variety of Gram-negative and Gram-positive bacterial species (Cook and Dunny, 2014), through conjugative (Ghigo, 2001) as well as Non-conjugative mechanisms, and, conversely, biofilms were reported to favor plasmids transfer, resulting in an increase of genetic exchange between bacteria, including antibiotic resistance genes (Van Meervenne et al., 2014). Plasmids are present in all *B. cereus, B. thuringiensis* and *B. anthracis* strains, in number, not including copies, ranging from 1 to 13, and in size ranging from 2 to almost 500 kb (Rasko et al., 2005; Reyes-Ramirez and Ibarra, 2008). Strains of these species also harbor integrated or Non-integrated temperate prophages (Rasko et al., 2005). While mobile genetic elements play a key role in the adaptation of *B. cereus* and related species to their specific environment, data on their involvement in biofilm formation or on the role of biofilms in their transfer are scarce for this group of bacteria. The role of plasmids in biofilm formation have not been considered until now, although there are indications that large pXO1-like plasmids contained in periodontitis or emetic strains might be involved in the specific behavior of these strains regarding this phenotype. Indeed, addition to the culture medium of cereulide, the product of the *ces* locus located on the pCER270 emetic strains pXO1-like plasmid, promotes the formation of biofilm (Ekman et al., 2012). Conversely, phages were shown to act on biofilm formation. The GIL01 and GIL16 prophages of the *tectiviridae* family, present as linear plasmids in *B. thuringiensis* strains, negatively affect biofilm formation and sporulation, and enhance swarming motility (Gillis and Mahillon, 2014). In *B. anthracis*, prophages of different families (*siphoviridae, myoviridae*, or *tectiviridae*) could either inhibit sporulation (Wip4, Wip5, Frp1), or induce this phenotype (Wip1, Wip2, Frp2) in culture conditions where spore formation does not usually occur—for example absence of aeration (Schuch and Fischetti, 2009). The lysogenic strains containing one of these phages displayed an increased production of cell-surface exopolysaccharides and an enhanced production of biofilms at the air-liquid interface in BHI culture medium (Schuch and Fischetti, 2009). The phages effect on the ability to produce exopolysaccharides or biofilms was the result of a prophage-chromosome dialog mediated by a sigma-factor-like regulator encoded in the prophage sequence (Schuch and Fischetti, 2009).

## Structure and properties

Data related to the biofilm structure are scarcely available in *B. cereus*. Although the *B. cereus* biofilm macrostructure has been described, the distribution in the biofilm of the different bacterial subpopulations or its morphogenesis are unknown, even more in the case of multispecies biofilms. Biofilm properties include adhesion to surfaces (which is dealt with in the part 5- Biofilm control in the food environment, of this review) and resistance to stresses. They also include the ability of the biofilm to produce spores, a property which add to the problems induced by the biofilm persistence.

### Structure

The *B. cereus sensu lato* floating pellicle displays differences in its architecture with the one produced by *B. subtilis*. The *B. subtilis* floating pellicle exhibits a high number of folds and do not bind to the recipient wall (Kobayashi, 2007a). In contrast, *B. cereus* biofilm, when formed at the air-liquid interface, includes a ring strongly sticking to the recipient wall, and the pellicle itself which displays protrusions instead of folds (Fagerlund et al., 2014). Wrinkles in the *B. subtilis* pellicle were shown to be a consequence of biomass extension, confined space, and elasticity of the pellicle, which is dependent from the extracellular matrix (Trejo et al., 2013). In *B. subtilis* colonies on agar plates, wrinkles forms preferentially where cell death occurs (Asally et al., 2012). The difference in the pellicle architecture between *B. cereus* and *B. subtilis* might be a consequence of the strong adhesion of the biofilm to the vessel walls in the former, and of the different polymers present in the matrix produced by the two species.

On immersed surfaces, *B. subtilis* and some *B. cereus* strains (see Section Ecological Aspects) are able to produce submerged biofilms. In the *B. subtilis* immerged biofilm, cells are organized in bundles which can, for some strains, protrude over the biofilm and form aerial structures at heights greater than 100 μm (Bridier et al., 2013). Few data are available on the structure of *B. cereus* immerged biofilm. The amount of biofilm formed in this condition was variable according to the strain, but a strain isolated from a food processing line produced, on stainless steel coupon, a thick and uneven biofilm with an aerial structure (Faille et al., 2014).

### Properties: Sporulation and resistance to stresses

The limited diffusion of nutrients and signal molecules within the matrix creates microenvironments in the biofilm, resulting in a heterogeneity of the bacterial population, which might include cells in the motile, virulent, necrotrophic, or sporulating states, as discussed in the Section Molecular and Physiological Aspects of this review. Sporulation rates in biofilms were highly variable and were dependent from the strain, the culture medium or the device used to form the biofilm (Table 1). Highest rates were obtained with strains isolated from the food environment and grown in poor media, with rates as high as 90%. Sporulation could occur in immerged biofilms although the rate of sporulation was increased when the biofilm was exposed to air or was let to dry (Ryu and Beuchat, 2005; Hayrapetyan et al., 2016), and was greater in the biofilm comparatively to the coexisting planktonic population (Hayrapetyan et al., 2015a). Stainless steel was more favorable to sporulation within the biofilm than polystyrene (Table 1). It was hypothesized that this result could be due to an increased iron availability on stainless steel coupons, as a consequence of corrosion (Hayrapetyan et al., 2015a). In addition to be suitable for sporulation, the biofilm confers to bacteria a protection against stresses. In biofilm, *B. anthracis* was from 40 (doxycycline) to 150 (ciprofloxacine) times more resistant to antibiotics than planktonic cells (Lee et al., 2007), and a multispecies biofilms containing *B. cereus* and *Pseudomonas fluorescens* was more resistant to antimicrobials than the biofilm of each species alone (Simoes et al., 2009).

**Table 1:** **Sporulation rates in biofilms after 48 h of incubation**.

**Strain**	**Subs^a^**	**Biofilm^b^**	**Device**	**Medium^c^**	**%Spore^d^**	**References**
Bc 98/4	SS	imm	Petri dish	TSB 1/10	87	Faille et al., 2014
Bc 5832	SS	imm	Petri dish	TSB 1/10	61	Faille et al., 2014
Bc D22	SS	imm	Petri dish	TSB 1/10	55	Faille et al., 2014
Ba Sterne	PS	air	96 wells plate	BHI	5	Lee et al., 2007
Bt 407	Glass	air	Glass tube	LBP	15^*^	Verplaetse et al., 2015
Bt 407	Glass	air	Glass tube	HCT	25^*^	Verplaetse et al., 2016
PAL25	PS	air	24 wells plate	Y1	91	Wijman et al., 2007
PAL25	PS	air	24 wells plate	LB	22	Wijman et al., 2007
ATCC10987	PS	air	24 wells plate	Y1	39	Wijman et al., 2007
ATCC10987	PS	air	24 wells plate	LB	10	Wijman et al., 2007
BC15	SS	air	12 wells plate	BHI	8	Hayrapetyan et al., 2015a
BC15	PS	air	12 wells plate	BHI	4	Hayrapetyan et al., 2015a
ATCC10987	SS	air	12 wells plate	BHI	2.5	Hayrapetyan et al., 2015a
ATCC10987	PS	air	12 wells plate	BHI	1	Hayrapetyan et al., 2015a
NIZO 4080	SS	air	12 wells plate	Y1	51	Hayrapetyan et al., 2016
NIZO 4080	PS	air	12 wells plate	Y1	38	Hayrapetyan et al., 2016
ATCC10987	SS	air	12 wells plate	Y1	13	Hayrapetyan et al., 2016
ATCC10987	PS	air	12 wells plate	Y1	3	Hayrapetyan et al., 2016

*Experiments were done at 30°C except for B. anthracis (37°) or for strains 98/4, 5832, and D22 of B. cereus (25°C)*.

a *Subs, substrate; SS, stainless steel; PS, polystyrene*.

b *Imm, immerged biofilm; air: biofilm at the air-liquid interface*.

c *Y1: defined culture medium*.

d *Percentage of spores relatively to the total number of colony forming units*.

* *These values represent the percentage of cells committed to sporulation instead of the actual percentage of spores*.

## Ecological aspects

In nature, bacteria live predominantly in biofilms rather than in a planktonic state (Costerton et al., 1995), and this observation is likely to stand also for *B. cereus* or *B. thuringiensis*. Consequently, biofilms are expected to be a key element for the adaptation of these species to their biotopes and to their biocenosis. However, *B. cereus* and its close relatives are found in a high diversity of biotopes, which questions the role that biofilm formation, in addition to other physiological properties, would play for their fitness to specific environments.

### Biofilm formation among *B. cereus* strains

Although biofilms are suspected to be involved in strains adaptation to their specific environment, there is a considerable variation in the ability to produce biofilms among isolates of *B. cereus* and *B. thuringiensis*, and no correlation was found between this ability and the origin (food poisoning, clinical, or environmental) of the strain (Wijman et al., 2007; Auger et al., 2009; Kuroki et al., 2009; Kamar et al., 2013; Hayrapetyan et al., 2015a). However, strains isolated from a specific niche, the oral cavity of periodontitis-diseased patients, were all unable to form biofilms (Auger et al., 2009), although these strains were isolated from dental plaques—which are biofilms. While unexpected, this result looks coherent since periodontal strains of *B. cereus*, as secondary colonizers of the dental plaque, do not need to initiate biofilms. Another interesting finding from prevalence studies is the observation that about 50% of *B. cereus* strains isolated from various food preparations produced less biofilms after 48 h than after 24 h of incubation (Hayrapetyan et al., 2015a), a proportion also found in emetic strains (Auger et al., 2009), which are frequent food contaminants (Ehling-Schulz et al., 2015). In contrast, only a minor proportion (less than 15%) of *B. cereus* strains isolated from blood samples (Kuroki et al., 2009), from the environment, or of *B. thuringiensis* strains (Auger et al., 2009) showed a drop in the biofilm biomass after 24 h of culture. This decrease can be explained by a massive emigration of biofilm cells. When back to the planktonic state, reverting cells will be able to create new biofilms and to spread the colonized area. Therefore, combined with their resistance to cleaning procedures (see the “*Bacillus* biofilms and their control in the food environment” section below), this property would confer food isolates the ability to persist and thrive in the food production lines.

Prevalence studies also revealed that the biomass of biofilms produced on stainless steel by *B. cereus* in LB or in a defined medium (Y1) is greater when they are formed at the air-liquid-solid interface than on submerged surfaces (Wijman et al., 2007). In BHI medium, only one strain, out of 23 isolates from food products, was able to form a submerged biofilm on polystyrene or on stainless steel coupons (Hayrapetyan et al., 2015a). Consequently, the property to form submerged biofilms appear to be rare among *B. cereus* strains. In the food industry production units, air-liquid interfaces are found in tanks while pipes are mostly in a flooded state. One would expect that the proportion of strains able to produce submerged biofilms would increase in isolates sampled from pipes when compared to isolates from tanks or to other isolates—although we have no data to support this expectation. It would be interesting to proceed to this comparison, since the ability to produce submerged biofilms affect *B. cereus* persistence within the food processing lines.

### *B. cereus* role in multispecies biofilms

Most biofilms found in natural environments include several bacterial species. *B. cereus* or *B. thuringiensis* make no exception to this observation and are found, when in biofilms, in association to other microorganisms. Multispecies biofilms are often described as cooperative consortiums where each partner contributes to the community resilience and development (Davey and O'toole, 2000). For example, periodontitis strains of *B. cereus* are found in the dental plaque (Rasko et al., 2007), which is one of the best studied multispecies biofilms. The dental plaquee is located at the tooth-gum interface and is a severe illness leading, ultimately, to gum bleeding, ligaments digestion and loosening and loss of teeth. Bacteria build the dental plaquee in a precise sequence, where pioneer species such as *Streptococcus mutants* bind first to the teeth enamel, followed by secondary colonizer species which bind to pioneer species or to themselves through a co-aggregation process (Kolenbrander et al., 2006). Secondary colonizers benefit from biofilm settlement by primary colonizers and, in turn, might contribute to the biofilm survival and growth. Indeed, *B. cereus* is able to shift the pH of a *Streptococcus mutants* biofilm from acidic to neutral values and in this way contributes to the biofilm pH balance (Sissons et al., 1998). It can also strongly participate to host tissues digestion owing to the numerous degradation enzymes which it secretes (Gohar et al., 2002) and which are present in the biofilm matrix (Karunakaran and Biggs, 2011). Likewise, *B. cereus* strains isolated from multispecies biofilms settled in paper machines were strong producers of exopolysaccharides (Ratto et al., 2005) and could therefore contribute actively to the biofilm development.

The integration of *B. cereus* vegetative cells can also occur in the depth of a Pre-existing biofilm, thanks to the high motility of these cells, which are able to create channels in the matrix and reach deep areas in the biofilm (Houry et al., 2010). Interestingly, *B. cereus* and *B. thuringiensis* secrete a number of bacteriocins (Ahern et al., 2003; Risoen et al., 2004; Oscariz et al., 2006), which, when produced within the integrated biofilm, could lead to drastic changes in the balance of bacterial biofilm populations. For example, a *B. thuringiensis* strain engineered to produce lysostaphin could invade and replace a *Staphylococcus aureus* biofilm native population (Houry et al., 2012), which clearly indicate that inter-species competition could occur within biofilms. Another example of competition between bacterial species within a natural biofilm is found in the pretreatment filters of water reclamation systems. These filters contain zeolite stones on which multispecies biofilms can grow. The *B. cereus* strains found in these biofilms are able to degrade the Gram-negative bacteria quorum sensing signal AHL (acylhomoserine lactone; Hu et al., 2003), interrupting the communication of their cohabitants and thus conferring a competitive advantage to *B. cereus*.

### Biofilms in soil, plants, and invertebrates

The environment is likely to be a major source of food contamination by microorganisms which can live in biofilms on plants or in the soil. *B. cereus* or *B. thuringiensis* are often described as saprophytic species whose natural habitat would be the soil (Vilain et al., 2006), from which they can easily be sampled (Vilas-Boas et al., 2002; Anjum and Krakat, 2016) and in which they can persist for long periods (Hendriksen and Carstensen, 2013). Interestingly, a number of *B. cereus* strains could multiply and form biofilm-like structures when cultivated in a liquid topsoil extract—but not in LB (Vilain et al., 2006), suggesting that some soil components are required to induce the formation of biofilm by *B. cereus* in the culture conditions used. However, not all soils can support *B. cereus* or *B. thuringiensis* growth, since an asporogenic strain of *B. thuringiensis* could not survive in a sterilized soil (Vilas-Boas et al., 2000), and it was speculated that the invertebrate gut rather than the soil might be the main ecological niche of these species (Jensen et al., 2003). *B. cereus* and *B. thuringiensis* were found in the gut of insects (Visotto et al., 2009), earthworms (Hendriksen and Hansen, 2002), nematodes (Schulte et al., 2010; Ruan et al., 2015), and isopods—which are terrestrial crustaceans (Swiecicka and Mahillon, 2006). In the intestine of insects and isopods, *B. cereus* forms filamentous structures described as “Arthromitus,” which proved to be chains of dividing bacteria (Margulis et al., 1998). Long chains of *B. cereus* or *B. thuringiensis* vegetative cells are typically found in biofilms, which suggests that these species can form biofilms in the gut of insects or isopods—and probably in the gut of other invertebrates as well.

In addition to the invertebrates gut, *B. cereus* is found in the rhizosphere and in the mycorrhiza of plants. When present in these subterranean structures, *B. cereus* can protect the plant from fungal attacks. For example, *B. cereus* UW85 produces zwittermicin A and kanosamine, both fungistatic molecules being suspected to contribute to the suppression of damping-off disease of alfalfa caused by *Phytophthora medicaginis* (Silo-Suh et al., 1994). Another strain of *B. cereus* (strain 0–9) isolated from roots of wheat cultures, was able to induce a reduction of 31% of the disease caused by the fungal pathogen *Rhizoctonia cerealis*, the agent of wheat sharp eyespot (Xu et al., 2014). A mutant of this strain obtained by random mutagenesis and selected for defective biofilm formation was unable to colonize wheat roots and to control the fungal disease (Xu et al., 2014). *B. cereus* is therefore likely to colonize plant roots through biofilm formation. This hypothesis is supported by the finding that, in *B. subtilis, tasA*, a gene required for biofilm formation which paralog is also required for biofilm formation in *B. cereus* (Caro-Astorga et al., 2015), is needed for the colonization of *Arabidopsis thaliana* roots (Lakshmanan et al., 2012). *B. cereus* can also be associated with plants through the mycorrhiza. It was, for example, isolated from *Glomus irregulare* spores sampled from the rhizosphere of *Agrotis stolonifera* growing in a natural stand (Lecomte et al., 2011) and was shown to form biofilms on the hyphae of *Glomus* sp. (Toljander et al., 2006). The arbuscular myccorhizal fungi are plant roots symbionts which mycelial network can explore soil volumes much larger than the roots themselves (Lecomte et al., 2011).

These data are summarized in the model depicted Figure 3, in which *B. cereus* and *B. thuringiensis* growing as biofilms in the topsoil would contaminate germinating plants, leading to biofilms on the rhizosphere and to spores on the phylloplane. Invertebrates feeding on roots (nematodes), soil organic matter (earthworms), vegetal debris (isopods), or leaves (caterpillars) would be infected by these bacteria, which could behave as commensals or as pathogens and settle as biofilms in their host gut. Invertebrates, through their mobility, could disseminate the bacteria in the environment and, upon death, contaminate back the topsoil, thus initiating a new cycle. Biofilms of *B. cereus* settled in soils and on plants could then contaminate raw food materials.

### The case of *B. anthracis*

Formation of biofilms by *B. anthracis* in the environment is controversial. *B. anthracis* does not need to produce biofilms for its infective cycle in mammals. Its spore is the infective agent, its toxins are extremely efficient and it is protected against the host immune defenses by a capsule. After the host death, *B. anthracis* multiply within the host, sporulate, and the spores are finally released into the environment at the host death spot. It is believed that the spores can survive in the soil for a long time, keeping their full infective properties, until their uptake by a new host. Yet, it has been argued that a multiplication step would be required to explain how slow the spore decay in soil is. Indeed, multiplication was observed in soil on plant roots, where *B. antthracis* formed long chains reminiscent of the bacterial chains found in biofilms (Saile and Koehler, 2006). *B. anthracis* can also produce biofilms in static and in flow conditions (Lee et al., 2007; Schuch and Fischetti, 2009). It expresses the regulators required for biofilm formation and at least a part the proteic components of the biofilm matrix (Pflughoeft et al., 2011), and can sporulate in biofilms (Lee et al., 2007). In addition, *B. anthracis* can colonize the earthworm gut for long periods (Schuch and Fischetti, 2009) and is found in flies and mosquitoes (Turell and Knudson, 1987), although only short-term colonization of flies gut was observed (Fasanella et al., 2010). While these data support a multiplication of *B. anthracis* outside its mammal host, further observations and experiments are required to determine if the model displayed **Figure 5** apply to this bacterium.

## Biofilms control in the food environment

*Bacillus* strains, including strains from the *B. cereus* group, can be isolated from endemic biofilms in various environments such as paperboard production or hospitals (Kolari et al., 2001; Ohsaki et al., 2007; Kuroki et al., 2009), but also food and beverage industries (Evans et al., 2004; Gunduz and Tuncel, 2006; Storgards et al., 2006; Marchand et al., 2012). The presence of biofilms containing *B. cereus* is a great concern for food industry settings such as fresh products, poultry, dairy, and red meat processing, and they are a potential source of recurrent cross-contamination and Post-processing contamination of finished products, sometimes resulting in food spoilage or foodborne illness (Rajkovic et al., 2008). The contamination of food processing lines by *B. cereus* biofilms could therefore be a serious public health risk, especially in foods that undergo mild processing such as minimally heat–treated foods (Tauveron et al., 2006). This risk must be given full attention since the total annual cost caused by *B. cereus* and *Staphylococcus aureus* in food illness is estimated at $523 million in the United States (Bennett et al., 2013).

### *B. cereus*, a food spoilage agent

As underlined above, the presence of biofilms in the food industry can result in food spoilage. Indeed, *B. cereus* strains produce extracellular proteases and lipases resulting in food degradation and spoilage, like sweet curdling and bitterness of milk sour taste, decreasing the shelf life of the product and therefore resulting in significant economic loss to food producers (Fromm and Boor, 2004; Flach et al., 2014). Even if present in raw milk at low concentration, *Bacillus* sp. become dominant after long periods of storage at a temperature of 10°C (which is often the case in shops), or when produced in improved technological conditions (Samarzija et al., 2012). Consequently, *Bacillus* spp. are today considered the main microbial causes for the spoilage of milk and milk products, and the main reason for significant economic losses in the dairy industry (Meer et al., 1991; Brown, 2000). It is estimated that the dairy industry has losses of up to 30 % due to spoilage and reduced product quality caused by psychrotrophic bacteria, including *Bacillus* sp. (Samarzija et al., 2012).

### Biofilms in food environments

**Figure 4:**
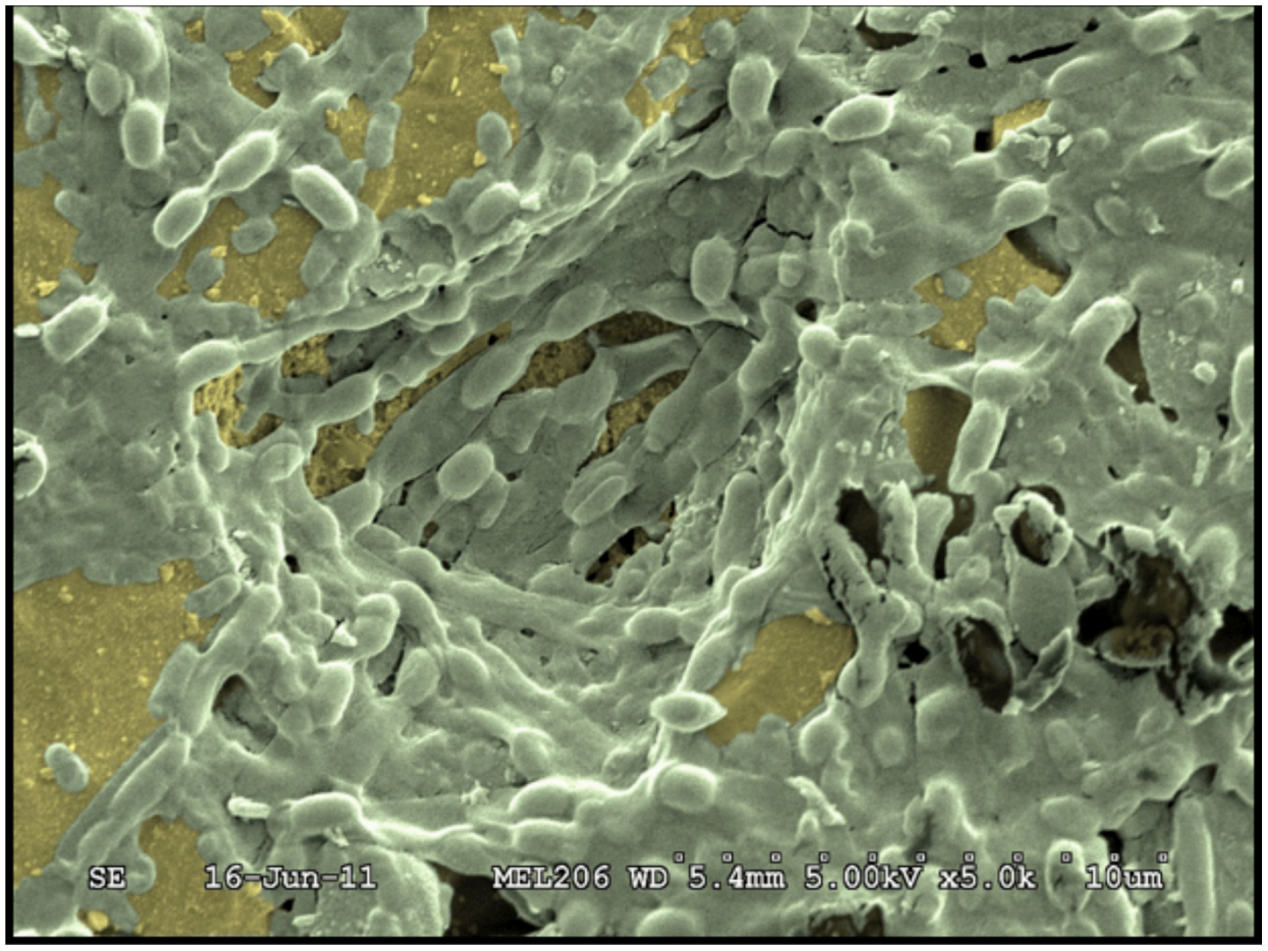
**Observation by scanning electron microscopy of a mixed biofilm formed by two strains: *B***. ***cereus*** 98/4 and *Comamonas testosteroni* CCL24 (Faille et al., 2014).

**Figure 5:**
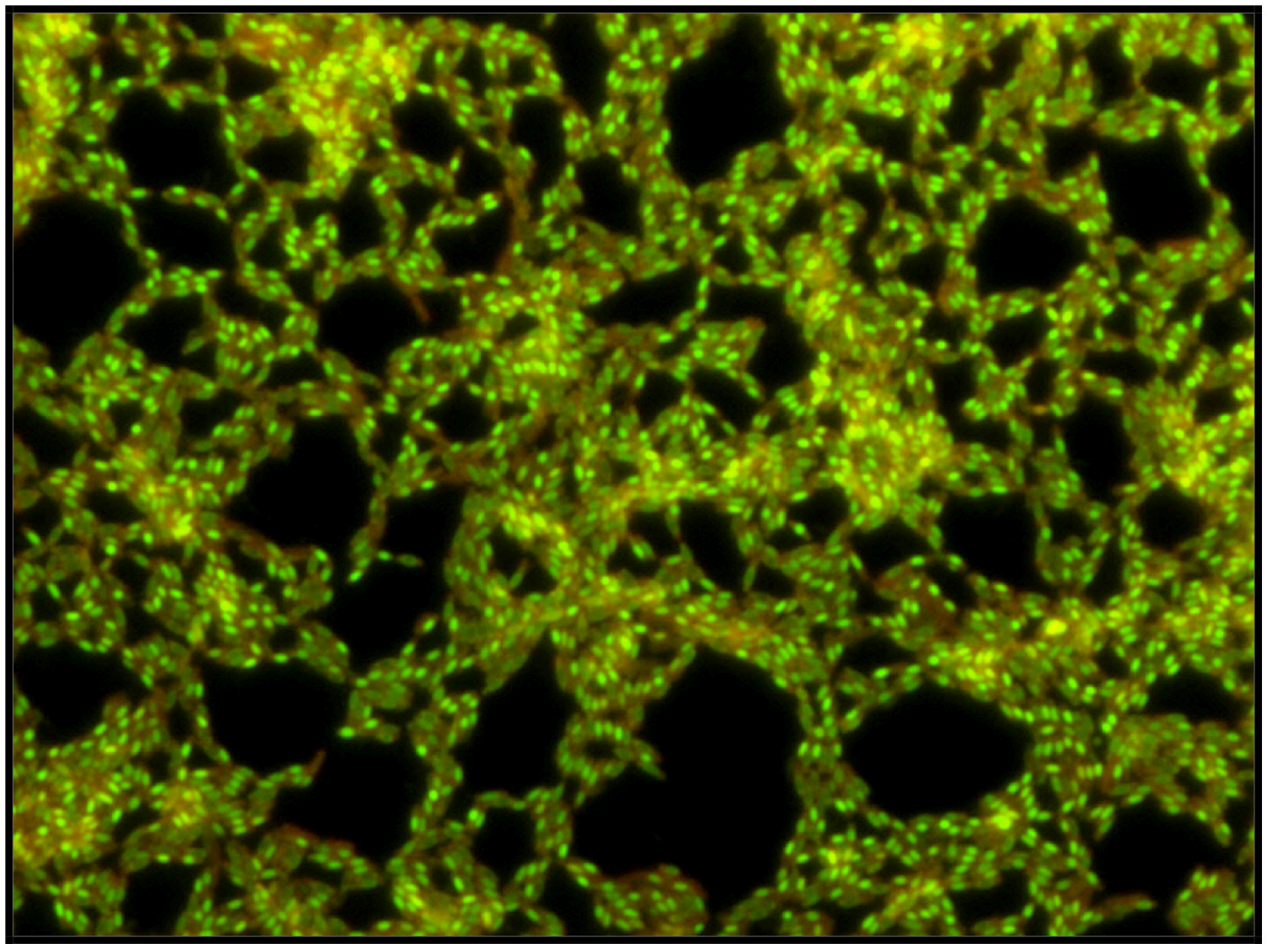
**Microscopic images of a *B. cereus* biofilm grown for 48 h in TSB 1/10**. Observation by epifluorescence after staining with the Live/Dead stain (magnification × 400). Endospores produced within the biofilm are stained in green, cells are stained in orange-green.

In food environments, *Bacillus* biofilms are found on every food contact surfaces of open or closed equipment, such as conveyor belts, pasteurizers, evaporators, filling machines, storage tanks, but also on cleaning and handling tools (Christison et al., 2007). Depending on the species or the strain, surfaces of cold rooms and equipment of processes lines where elevated temperatures prevail could be contaminated by *Bacillus* biofilms (Sharma and Anand, 2002a; Kolari et al., 2003; Evans et al., 2004; Gunduz and Tuncel, 2006; Kumari and Sarkar, 2014). In fact, *Bacillus* spores or biofilms are capable of contaminating every surface commonly found in food-industry plants, including inert surfaces such as stainless steel surfaces (Faille et al., 2014), plastics or rubber (Mettler and Carpentier, 1997), but also surface of vegetables (Elhariry, 2011). Moreover, *Bacillus* strains are able to form biofilms both under static and flow conditions, and thick biofilms of *B. cereus* would particularly develop at the air-liquid interface (Wijman et al., 2007). Along food processing lines, *B. cereus* is often found in association with other bacterial species to form mixed biofilms (Figure 4) where high levels of *Bacillus* isolates have sometimes been reported (Mattila et al., 1990). For example, percentages as high as 25% of *Bacillus* sp. isolates (including *B. cereus* isolates) have been found in dairy processing industries (Sharma and Anand, 2002c). In addition, sporulation occurs within biofilms (Figure 5) on food contact surfaces (Storgards et al., 2006), sometimes at very high levels (De Vries et al., 2004; Faille et al., 2014), suggesting a potentially significant role for biofilm-derived spores in contamination of food with *Bacillus* spp. (Scott et al., 2007).

### Biofilms control

In food plants, disinfection of processing lines (e.g., pipes, heat-exchangers, valves tanks) is preceded by a cleaning step, involving alkali or other cleaning agents. Cleaning and sanitation procedures are set up to guarantee the detachment of organic and inorganic contaminations, disinfection of the cleaned surface and elimination of the residues of the sanitation agents (Vlkova et al., 2008). Unfortunately, the detachment of spores and biofilms but also of food residues in the food processing environment is critical since they often accumulate in areas which are difficult to clean, e.g., crevices, valve, gaskets, and dead ends (Czechowski, 1990; Austin and Bergeron, 1995; Sharma and Anand, 2002b). Of particular concern is the increased resistance of biofilms, compared with bacteria in a free-living environment, to disinfection processes. For example, two widely-used sanitizers, a quaternary ammonium compound and sodium hypochlorite, did not effectively inactivate the adherent single cells and biofilms of *B. cereus* at concentrations able to induce a reduction in CFU/ml of more than 5.0 log of their planktonic counterparts. Furthermore, the efficacy of both disinfectant was even lower when biofilms were formed on milk Pre-soiled stainless steel (Peng et al., 2002). Adherent *Bacillus* spores also exhibit a greater resistance to high temperature and disinfectant than spores in suspension (Sagripanti and Bonifacino, 1999; Faille et al., 2001; Kreske et al., 2006a). Indeed, residual *Bacillus* contamination of equipment surfaces after cleaning and/or sanitizing procedures was detected at different points on milk pasteurization lines and on the surface of the packaging machine (Mattila et al., 1990; Sharma and Anand, 2002b; Salustiano et al., 2009). Hence, considering the difficulty in inactivating adherent *Bacillus* spores and biofilms, cleaning the biomass from the surfaces is fundamental for controlling biofilm development.

### Cleaning-in-place protocols

The cleaning-in-place (CIP) protocols used to clean processing lines without dismantling or opening of the equipment, vary according to industries or the food chain and the residues that need to be cleaned, although caustic and acid cleaning has remained the standard method used in many food processing industries. Both chemical (cleaning agents) and mechanical (shear stresses) actions are supposed to play a major role on soil removal. However, the effectiveness of CIP regimes against *B. cereus* biofilm has not been extensively reported. In the food industries, CIP regimes frequently involve a 60°C cleaning alkali wash (mainly sodium hydroxide), followed by an acid (mainly nitric acid) wash disinfection step (Bremer et al., 2006), but a reduction of viable spores by only 40% has been reported (Andersson et al., 1995). In the case of *Bacillus* biofilms, relatively low efficiency of the reference CIP regime (1% NaOH at 65°C for 10 min—water rinse—1% HNO_3_ at 65°C for 10 min—water rinse) has been reported, but the removal would be improved by increasing the concentration of NaOH or the duration of the cleaning procedure (Flint et al., 1997; Bremer et al., 2006; Kumari and Sarkar, 2014).

### Mechanical and chemical cleaning

In order to better understand the mechanism of spore and biofilm detachment during CIP, the respective role of rinsing vs. cleaning (mechanical and chemical forces) in the detachment of *Bacillus* biofilms and spores was investigated. When the *B. cereus* biofilm was formed on milk Pre-soiled stainless chips (Peng et al., 2002) or at different shear stresses (Lemos et al., 2015), a rapid population decrease occurred during the first 5 min whatever the detachment conditions, and no further removal was observed for longer times, either in terms of vegetative cells or spores, even if the amount of detached biofilm was significantly higher in the presence of cleaning agents. Similar observations have been reported when *B. cereus* biofilm was formed on milk Pre-soiled stainless chips (Peng et al., 2002) or at different shear stresses (Lemos et al., 2015). Further works, performed on spores from the *B. cereus* group, demonstrated that during a CIP, chemical action plays a major role in the detachment of adherent spores, while mechanical action is poorly effective (less than 90% decrease in the number of adherent spores at wall shear stresses of 500 Pa, whatever the strain; Faille et al., 2013). Spores produced in biofilms showed greater resistance to detachment than the complete biofilms on inert surfaces (Faille et al., 2014) and on vegetables (Elhariry, 2011).

If the contaminated areas are allowed to dry before cleaning, e.g., in half-filled tanks or pipes or on open surfaces, the sporulation level would increase within *Bacillus* biofilms (Hayrapetyan et al., 2016) and the resistance to shear of attached spores increase concomitantly (Nanasaki et al., 2010). The increase in resistance to detachment is particularly noteworthy for long times and/or high temperature of drying (Faille et al., 2016).

In order to improve the efficiency of cleaning procedures, some industrialists opted to develop enzymatic cocktails effective against biofilms found in food processing plants, which are known to poorly respond to traditional cleaning procedures. The enzymes offer major advantages over traditional cleaning solutions, e.g., low toxicological risk and ecological risk, ease of rinsing external residues and compatibility with different surface material. Many products are nowadays commercially available, essentially for medical use. Some of the commercialized cocktails have proven their efficiency against biofilms produced by *B. cereus, B. mycoides* or *B. flavothermodurans*, and also against *B. cereus* adherent spores (Langsrud et al., 2000; Parkar et al., 2004; Lequette et al., 2010). These enzymatic “detergents” being more expensive than conventional products, their use is proposed as a complementary solution to current cleaning procedures.

Spores and, to a lesser extent, vegetative cells embedded in a *B. cereus* biofilm are protected against inactivation by the sanitizers commonly used to control foodborne pathogens, such as chlorine and hydrogen peroxide, which are easy to handle, inexpensive, and are soluble in water and relatively stable over a long storage time. For example, hydrogen peroxide or peracetic acid show little activity on adherent *B. subtilis* and *B. cereus* spores (Faille et al., 2001; Dequeiroz and Day, 2008). At higher temperatures and longer exposures, a significant reduction in *B. cereus* viable counts would be observed, but it is not suitable for practical disinfection due to corrosion and toxicity (Langsrud et al., 2000; Dequeiroz and Day, 2008). However, although the peroxygen-based disinfectants are not sporicidal alone, the use of NaOH 1% (typically used at 0.5–2% in the food and beverage industries) or of an enzymatic cocktail would sensitize *Bacillus* spores to the action of these oxidative disinfectants (Langsrud et al., 2000). The activity of sodium hypochlorite on *B. cereus* spores on surfaces and in field trials is also limited (Te Giffel et al., 1995). Indeed, although hypochlorite solutions are more stable above pH 9.5, they are only efficient at neutral or acidic pH (Sagripanti and Bonifacino, 1999). However, a marked synergistic effect between both was described on the efficacy to reduce spore counts on contaminated surfaces (Dequeiroz and Day, 2008). The same phenomenon was observed with biofilms produced in immersed conditions or exposed to air (Ryu and Beuchat, 2005). Furthermore, chlorine dioxide was less effective than chlorine in killing *Bacillus* spores on stainless steel, mainly in the presence of organic soil (Kreske et al., 2006a) and injured *B. cereus* cells were sometimes seen to recover overnight (Lindsay et al., 2002). Within biofilms, spores were more resistant to chlorine and chlorine dioxide than the vegetative cells (Kreske et al., 2006b).

### Control of multispecies biofilms including *B. cereus*

The control of mixed species biofilms including *B. cereus* and other *Bacillus* species has also been investigated. For example, the efficiency of sodium hypochlorite and iodophor, commonly used in the beverage and dairy industries, has been studied in different segments of pasteurization lines (Sharma and Anand, 2002b). Results from this study suggest that sodium iodophors were in some cases more efficient than sodium hypochlorite in inactivating biofilms and that the latter treatment was affected by the constitutive microflora or by spatial heterogeneity of biofilms. However, biofilms were still detected on the different areas even after CIP and iodophor treatment. Since iodophors are much less active against spores than hypochlorite, one can hypothesize that the residual biofilms following treatment with iodophors would be largely composed of *Bacillus* spores. A laboratory work on dual biofilms (*B. cereus* and *P. fluorescens*) showed that dual biofilms are characterized by an increased stability to shear stress and are more resistant to a quaternary ammonium compound (QAC), cetyltrimethylammonium bromide, and glutaraldehyde solutions (sanitizers commonly used in the medical field) than each single species biofilm (Simoes et al., 2009). Once more, a significant proportion of the population of both bacteria remain in a viable state after exposure to antimicrobials. The presence of residual bacterial population after treatment by QACs, also frequently used in food-processing industries, could encourage the development of resistance among food-associated bacteria, as already observed in Gram-negative bacteria and *Enterococcus* spp. (Sidhu et al., 2002).

## Concluding remarks

In the last decade, a number of studies have shown that although *B. cereus sensu lato* biofilms looked the same as the *B. subtilis* ones, there are quite different in several aspects. These studies brought a huge improvement to our understanding of how *B. cereus* biofilms are built, what is their contribution to the bacterium lifestyle, or how to get rid of them when required. Still, a number of issues stay unresolved or has been brought to light by recent findings. While the role of the TasA-like proteins in the biofilm matrix has been confirmed, the duplication of their genes asks the question of their role in the biofilm formation and in the adaptation of the bacterium to its environment or to its host. Similarly, the genetic determinants required for the building of the polysaccharidic part of the matrix remains a mystery, as well as the regulation of their production and the role of the large *epsA-O* -like polysaccharidic locus, since this locus does not seems to be involved in biofilm formation. The mechanisms through which eDNA, which was found in high quantities in the *B. cereus* biofilm matrix, is released remains unknown. The possible involvement of programmed cell death (PCD) in this release as well as in the shaping of the biofilm architecture, and the connection of its regulation to the regulation of biofilm formation represent other exciting issues in the forthcoming work on *B. cereus* biofilm formation. The impact of plasmids, which are known to play a major role in *B. cereus sensu lato* pathogenesis, on biofilm formation, and the mechanism through which plasmids act on this phenotype is still to be determined. Regarding pathogenesis, the presence and the evolution of biofilms *in vivo* has not been yet established, nor has been their exact contribution to the bacterium virulence. Another important issue is relative to the role of biofilms in the *B. cereus sensu lato*, including *B. anthracis*, survival and growth in the soil environment. Finally, the traditional hygiene procedures used in the food industry have revealed their limit in the control of surface contamination with *Bacillus* spores and biofilms. If we consider that *B. cereus* and other species can act as spoilage organisms and pathogens, these surface contaminations are still of concern in the food industry. This problem is thus far from being resolved and there are many questions that remain to be addressed concerning the different approaches to manage the surface hygiene and limit the risks to consumers.

## Author contributions

All authors listed, have made substantial, direct and intellectual contribution to the work, and approved it for publication.

## Funding

Researches were funded by the Agence Nationale pour la Recherche (ANR, France), Campus France, the University St Joseph of Beirut and the Conseil National de la Recherche Scientifique (CNRS-L, Lebanon). These agencies had no role in this work (study design, data analysis, manuscript writing)

### Conflict of interest statement

The authors declare that the research was conducted in the absence of any commercial or financial relationships that could be construed as a potential conflict of interest.
